# Preparation and Pharmacokinetic Study of Daidzein Long-Circulating Liposomes

**DOI:** 10.1186/s11671-019-3164-y

**Published:** 2019-10-15

**Authors:** Qiao Wang, Wenjin Liu, Junjun Wang, Hong Liu, Yong Chen

**Affiliations:** Hubei Province Key Laboratory of Biotechnology of Chinese Traditional Medicine, Hubei Collaborative Innovation Center for Green Transformation of Bio-resources, National &Local joint Engineering Research Center of High-throughput Drug Screening Technology, Hubei University, Wuhan, 430062 China

**Keywords:** Daidzein, Long-circulating liposome, Encapsulation efficiency, In vitro release, Pharmacokinetics

## Abstract

In this study, daidzein long-circulating liposomes (DLCL) were prepared using the ultrasonication and lipid film-hydration method. The optimized preparation conditions by the orthogonal design was as follows: 55 to 40 for the molar ratio of soybean phosphatidylcholine (SPC) to cholesterol, 1 to 10 for the mass ratio of daidzein to total lipid (SPC and cholesterol) (w:w), the indicated concentration of 5% DSPE-mPEG2000 (w:w), 50 °C for the hydration temperature, and 24 min for the ultrasonic time. Under these conditions, the encapsulation efficiency and drug loading of DLCL were 85.3 ± 3.6% and 8.2 ± 1.4%, respectively. The complete release times of DLCL in the medium of pH 1.2 and pH 6.9 increased by four- and twofold of that of free drugs, respectively. After rats were orally administered, a single dose of daidzein (30 mg/kg) and DLCL (containing equal dose of daidzein), respectively, and the MRT_0−*t*_ (mean residence time, which is the time required for the elimination of 63.2% of drug in the body), *t*_1/2_ (the elimination half-life, which is the time required to halve the plasma drug concentration of the terminal phase), and AUC_0−*t*_ (the area under the plasma drug concentration-time curve, which represents the total absorption after a single dose and reflects the drug absorption degree) of daidzein in DLCL group, increased by 1.6-, 1.8- and 2.5-fold as compared with those in the free group daidzein. Our results indicated that DLCL could not only reduce the first-pass effect of daidzein to promote its oral absorption, but also prolong its mean resident time to achieve the slow-release effect.

## Background

Daidzein is a natural compound found exclusively in soybeans and other legumes and structurally belongs to a class of compounds known as isoflavones. The pharmacological activity has been reported for daidzein in the prevention and therapy for cardiovascular disease [1], menopausal relief [2], osteoporosis [3], lowering the risk of some hormone-related cancers [4], and anti-inflammatory effect [5]. Due to the chemical structure, daidzein has very poor water solubility and lipid solubility and was mainly absorbed in the intestinal tract after oral administration and easy to metabolize forming glucuronic acid conjugate or sulfuric acid conjugate [6–10]. In order to improve its poor bioavailability, recent studies were focused on its novel drug delivery system, such as daidzein phospholipid complex [11], daidzein self-assembled micelle [12], and polylactic acid nanoparticle [13].

Liposome is an effective drug carrier system, which can encapsulate hydrophobic drugs, hydrophilic drugs, and drugs that interact with phospholipids [14, 15]. Due to its very good biocompatibility, liposomes can increase the intestinal permeability, reduce chemical and biological degradation, and reduce non-specific side effects of drugs [16]. However, the use of conventional liposomes cannot fully overcome their binding with serum components and uptake by mononuclear phagocyte system (MPS) [17]. To overcome such problems, long-circulating liposomes, modified with a hydrophilic or a glycolipid such as (polyethylene glycol) (PEG) or mono-sialoganglioside (GM1), have been developed in the past several years. The presence of PEG on the surface of the long-circulating liposomal carrier has been shown to form a layer of hydrophilic protective film, which can prevent the liposome interacting with a variety of components in the serum and consuming by phagocytes recognition [18]. Therefore, long-circulating liposomes can extend blood-circulation time by reducing MPS uptake and thereby improve the bioavailability of drugs [19, 20]. In this paper, the preparation method of daidzein long-circulation liposome (DLCL), as well as its in vitro release and pharmacokinetic characteristics in rats, was investigated. The results provide experimental basis for the clinical application of DLCL.

## Methods

### Materials

Soybean phosphatidyl choline (SPC) was purchased from Lipoid GmbH (Germany). Cholesterol and DSPE-mPEG2000 were purchased from AVT Pharmaceutical Co., Ltd. (Shanghai, China). HPLC-grade methanol and acetonitrile were purchased from TEDIA Company (USA). Chloroform and methanol (analytical grade) were obtained from Sinopharm Chemistry Reagent Co., Ltd. (Shanghai, China). The water was purified by Milli-Q® water purification system ((Millipore, USA). Daidzein (≥ 98% in purity) was purchased from Yuanye Biotechnology Co., Ltd. (Shanghai, China). Apigenin (internal standard, IS, ≥ 98% in purity) was purchased from Delge Pharmaceutical Technology Co., Ltd. (Nanjing, China). Phosphotungstic acid hydrate (analytical grade) was purchased from Macklin Co., Ltd. (Shanghai, China). Tween-80 and ethyl acetate were obtained from Sigma (Missouri, USA). Formic acid (MS-grade) was purchased from Fischer (USA).

### Animals

Ten male Sprague-Dawley rats (200–210 g) were purchased from the disease prevention and control center of Hubei province with the license number of SCXK (E) 2017–0012. The animal experiment was approved by the Ethics Committee of Hubei University, and complied with the guide for the care and use of laboratory animals.

### Preparation of Daidzein Long-Circulating Nanoliposome (DLCL)

Taking particle size and encapsulation efficiency (EE) as evaluation indexes, the orthogonal design of four factors and three levels (Table 3) was performed to optimize the best matching of the molar ratio of SPC to cholesterol (A), the mass ratio of the drug (daidzein) to the total lipid (SPC and cholesterol) (w/w) (B), hydration temperature (C), and ultrasonic time (D) at the condition of 5% content of DSPE-mPEG2000 [21, 22].

DLCL was prepared by thin film evaporation-sonication method described briefly as follows [21]: soybean phosphatidylcholine, cholesterol, DSPE-mPEG2000, and daidzein were dissolved in a round-bottomed flask with 10 mL chloroform-methanol (1:4, v/v) mixture. Under the conditions of vacuum and 40 °C (water bath), the mixture was dried to form a thin film in the rotary evaporation apparatus (RE-2000A, Shanghai Yi-Rong Biochemical Instrument Factory, China), and then hydrated with 20 mL ultrapure water by sonication (80 w) for 24 min in ice bath. The liposomal suspension was extruded three times by filtering through 0.45 μm and 0.22 μm microporous membrane in turn. The prepared DLCL solution was stored at 4 °C. Long-term preservation requires the addition of 3% sucrose (used as a lyoprotectant) to the DLCL suspension and freeze-drying preservation at − 20 °C.

### Determination of Daidzein in DLCL by HPLC

**Fig. 1:**
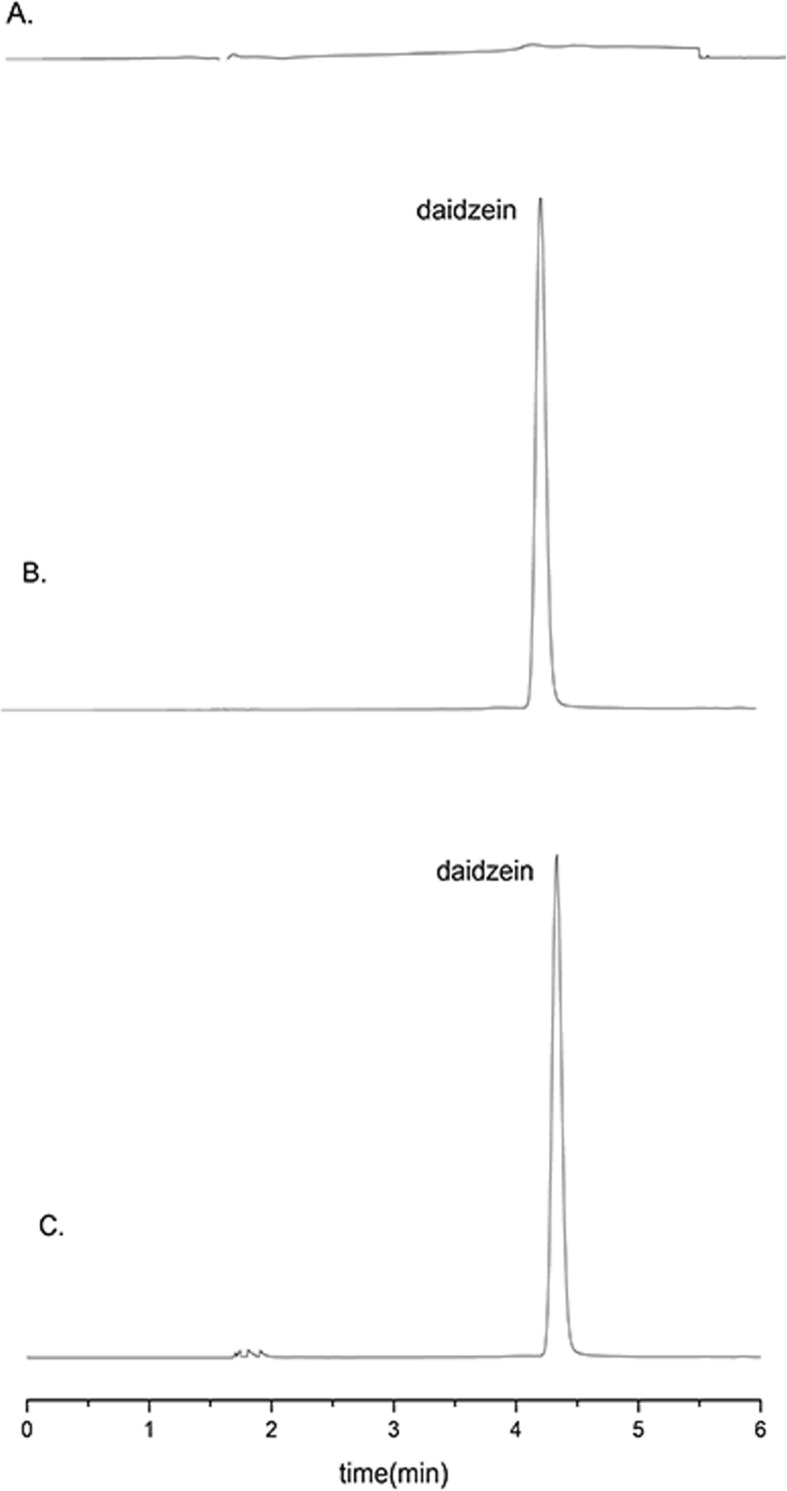
Typical chromatograms of blank liposomes (**a**), daidzein reference substance (**b**), and DLCL sample (**c**)

The column was a Phenomenex ODS analytical column (150 mm × 4.6 mm, 5 μm) connected to a guard column (30 mm × 10 mm, 3 μm) with the column temperature of 40 °C. The mobile phase consisted of 10 mM aqueous ammonium acetate solution (A) and methanol (B) with the gradient elution as follows: 0–3.0 min, 45% B to 80% B; 3.0–4.0 min, 80% B; 4.0–6.0 min, 80% B to 45% B. The flow rate was 1 mL/min. The detection wavelength was 240 nm. The injection volume was 10 μL. The linear range of daiazein was 0.313–50 μg/mL, and the regression equation was *y* = 35,461x + 1802.4, *R*^2^ = 0.9999. The retention time of daidzein is 4.30 min, and no interference from the DLCL formulation was found on the determination of daidzein (Fig. 1). The precision, reproducibility, stability, and sample recovery of the analysis method were strictly investigated and met the requirements of the quantitative analysis (Table 1).

**Table 1:** Precision, reproducibility, recovery, and stability of daidzein detected by HPLC method (*n* = 6)

Components	Concentration (ng/mL)	Precision (%, RSD)	Reproducibility (%, RSD)	Recovery (%)	Stability (%, RSD)
Daidzein	0.3125	1.18	0.83	96.0	2.17
	10	1.01	0.71	96.2	2.01
	50	0.96	0.59	96.7	2.03

### Prescription Screening by Orthogonal Test

Taking particle size and encapsulation efficiency (EE) as evaluation indexes, the orthogonal design of four factors and three levels was performed to optimize the best matching of the molar ratio of SPC to cholesterol (A), the mass ratio of the drug (daidzein) to total lipid (SPC and cholesterol) (w/w) (B), hydration temperature(C), and ultrasonic time (D) at the condition of 5% content of DSPE-mPEG2000 [21, 22].

### Diameter and Morphology of DLCL

The particle size and zeta potential of the prepared DLCL solution were measured by a laser particle size analyzer (Zetasizer Nano90, Malven Instruments Limited, Worcestershire, UK) at room temperature, 230 V and 50 HZ. The prepared DLCL solution was diluted 10 times with pure water and then taken up from the edge of the copper mesh with tweezers. After drying at room temperature and counter-staining by phosphotungstic acid aqueous solution (2%, w/v), the morphology of the completely dried DLCL was observed using a field emission transmission electron microscope (JEM-2100 (HR), JEOL Ltd., Tokyo, Japan) at acceleration 200 kV and transmitter LaB6.

### EE and Drug Loading of DLCL

The EE and drug loading of the prepared DLCL were assayed by the dialysis method stated as follows: (1) 500 μL of the prepared DLCL solution was added into a dialysis bag with the molecular weight cutoff of 8000–14,000 (BioSharp Sai-Guo Biotechnology Co., LTD), then tied the two ends of the dialysis bag tightly, and put the dialysis bag into 20 mL of water (dialysis medium). After vibrating with shaker for 12 h, 1 mL of the dialysis medium was taken and centrifuged at 12000 rpm for 10 min to determine the free drug concentration (*C*_1_) in the supernatant. (2) Five hundred microliters of the prepared DLCL solution and 2000 μL of methanol were mixed by vortexing for 15 min to destroy the liposomes and then centrifuged at 12000 rpm for 10 min to determine the total drug concentration (*C*_0_) in the supernatant. (3) Ten milliliters of the prepared DLCL solution (*V*_0_) was lyophilized to weigh the solid powder mass (*W*_0_). (4) The EE and the drug loading were calculated according to the formula stated as follows:

$$ \mathrm{EE}\left(\%\right)=\left({C}_0\hbox{-} {C}_1\right)/{C}_0\times 100,\kern1.5em \mathrm{Drug}\kern0.5em \mathrm{loading}\left(\%\right)={C}_0\cdotp {V}_0\cdotp \mathrm{EE}/{W}_0\times 100 $$

### In Vitro Drug Release

The in vitro release of DLCL and free daidzein was determined by dialysis method. The simulated gastric fluid was 0.1 mol/L HCl (pH 1.2) containing 0.5% Tween-80, and the simulated intestinal fluid was 25 mM PBS buffer (pH 6.9) containing 0.5% Tween-80. 0.5 mL of the prepared DLCL solution was added into a dialysis bag with a molecular weight interception of 8000–14,000 and placed in 20 mL of the two release media, respectively. The release test media were stirred (100 rpm) continuously at 37 °C. At the indicated time points (0, 0.5, 1, 2, 4, 6, 8, 10, 12, 24, 36, 48, 60, 72, 96, 120, and 144 h), aliquots of the sample (1 mL) were taken from the dialysate and then supplemented the same amount of fresh release media. One milligram per milliliter of daidzein (dissolved in DMSO) was used as a control and placed respectively in the above two release media for the same treatment. Daidzein was measured at each time point, and the cumulative release rate was calculated according to the formula:

$$ \mathrm{Cumulative}\kern0.5em \mathrm{release}\kern0.5em \mathrm{rate}\left(\%\right)=\left[{V}_1\times \left({C}_1+{C}_2+\dots +{C}_{i\hbox{-} 1}\right)+{V}_2\times {C}_i\right]/\left({V}_{\mathrm{o}}\times {C}_{\mathrm{o}}\right)\times 100 $$

In the formula, *V*_1_ was the sampling amount at each time point, *V*_2_ was the volume of the dialysis medium, *C*_1_~*C*_*i*_ was the concentration of daidzein measured at each time point, and *V*_0_ and *C*_0_ were the volume and concentration of DLCL added to the dialysis bag.

### Pharmacokinetics of DLCL in Rats

Ten male Sprague-Dawley rats were randomly divided into two groups (*n* = 5). One group was daidzein (suspended in 0.5% sodium carboxymethylcellulose) group, and another group was DLCL (dissolved in water) group. The doses of daidzein in the two groups were 30 mg/kg. Before the oral administration, rats were fasted for 12 h and free to drink water. After the rats were intragastrically administered, the daidzein group was taken 0.5 mL blood from the fundus venous plexus of each rat at 3 min, 5 min, 10 min, 15 min, 30 min, 45 min, 60 min, 1.5 h, 2 h, 3 h, 4 h, 6 h, 8 h, 10 h, 12 h, 24 h, and 36 h, respectively. Meanwhile, the DLCL group was taken 0.5 mL blood from each rat by the same way at 1 min, 3 min, 5 min, 10 min, 15 min, 30 min, 45 min, 60 min, 1.5 h, 2 h, 3 h, 4 h, 6 h, 8 h, 10 h, 12 h, 24 h, and 36 h, respectively. The blood sample was placed in a heparinized centrifuge tube and centrifuged at 4000 rpm for 10 min, and the plasma was taken to store at − 80 °C. The content of daidzein in the plasma sample was determined by the established LC-MS/MS method to obtain its drug-time curve in the tested rats. The pharmacokinetic parameters were calculated with non-compartment model by using DAS3.0 software (Professional Member of Chinese Pharmacology Society, Shanghai, China).

### Determination of Daidazein in Rat Plasma by LC-MS/MS

**Fig. 2:**
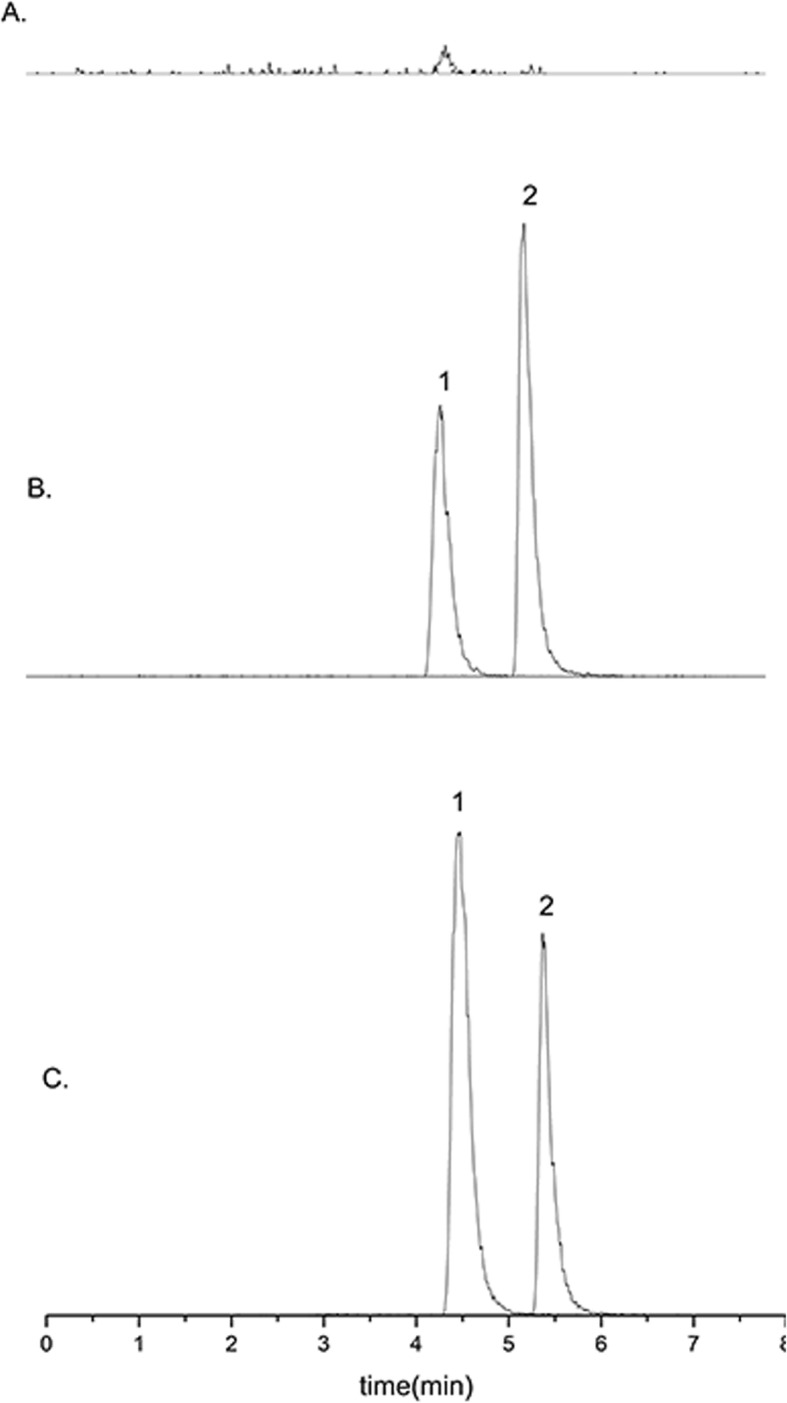
HPLC of blank plasma (**a**), daidzein + apigenin (internal standards) + blank plasma (**b**), and plasma sample (**c**). 1, daidzein; 2, apigenin (IS)

Rat plasma samples were pretreated as follows: rat plasma (50 μL), methanol (10 μL), internal standard (IS) apigenin dissolved in methanol (10 μL, 500 ng/mL), and 5% formic acid aqueous solution (100 μL) were mixed in a clean 1.5-mL test tube. Following briefly the vortex-mixing, 1.2 mL of ethyl acetate was added into the mixture. After shaking at room temperature for 5 min, the mixture was centrifuged at 12,000 rpm for 10 min. The resulting upper organic phase (1 mL) was transferred into another clean 1.5-mL test tube, evaporated and re-dissolved in the mobile phase (100 μL). The supernatant obtained by centrifuging at 12,000 rpm for 10 min was collected for LC-MS/MS analysis. The quantitative conditions of rat plasma samples are described below: GL Inertsustain C18 (100 mm × 2.1 mm, 3 μm) was connected to a Shim-pack Column Holder guard column (5.0 mm × 2.0 mm, 1.6 μm) at a column temperature of 40 °C; the gradient elution with the flow rate of 0.2 mL/min was performed using the mobile phase consisted of water (A)-methanol (B) in the conditions of 50% B to 80% B (0–2.00 min), 80% B (2.00–4.00 min), 80% B to 50% B (4.00–6.00 min), and 50% B (6.10–8.00 min). The injection volume was 10 μL. The negative ion multi-reaction monitoring mode (MRM) was used to detect the ion pairs of daidzein (*m*/*z* 253.0 → 224.15) and IS (*m*/*z* 269.00 → 117.05). The other conditions were stated as follows: ESI ion source, heating block temperature 400 °C, DL tube heating temperature 250 °C, atomizing gas (*N*_2_) volume flow 3.0 L/min, drying gas (*N*_2_) volume flow 15.0 L/min, and ion spray voltage − 4.5 V. The retention times of daidzein and internal standard (apigenin) were 4.5 min and 5.4 min, respectively, and the endogenous substances in plasma did not interfere with the determination of daidzein and internal standard (Fig. 2). Quantitative methodology was strictly investigated and met the requirements of quantitative analysis of biological samples (Table 2).

**Table 2:** Precision, matrix effect, extraction recovery and stability of daidzein, and IS detected by LC–MS/MS method (*n* = 6)

Components	Concentration (ng/mL)	Within-batch precision (%, RSD)	Between-batch precision (%, RSD)	Matrix effect (%)	Extraction recovery (%)
Daidzein	10	7.21	4.68	69.97	114.37
	500	6.48	6.32	87.64	93.21
	800	4.45	5.98	86.37	91.81
IS	100	–	–	84.86	96.59
Components	Concentration (ng/mL)	Stability (%, RSD)			
		Freeze and thaw three times	Placed at room temperature for 24 h	Freezing at − 20 °C for 5 days	
Daidzein	10	9.87	8.73	3.78	
	500	3.42	6.95	6.89	

### Statistical Analysis

Data are presented as mean ± standard deviation (SD). Statistical comparisons were made using one-way analysis of variance (ANOVA), followed by Tukey’s test. Values were considered statistically significant at *p* <  0.05.

## Results and Discussion

### Effect of Preparation Processes on Nanoliposome Characteristics

The orthogonal test design and test results are shown in Table 3, and the variance analysis results are shown in Table 4. Intuitive analysis (Table 3) showed that the order of influence of four factors on particle size was drug-lipid ratio > SPC-cholesterol ratio > hydration temperature > ultrasonic time, which had little effect on encapsulation efficiency. Analysis of variance (Table 4) showed the drug-lipid ratio; SPC-cholesterol ratio has a significant effect on particle size. The optimal preparation conditions for DLCL were A_1_B_1_C_2_D_1_, the ratio of SPC to cholesterol was 55:40, the ratio of drug to lipid was 1:10, the hydration temperature was 50 °C, and the ultrasonic time was 24 min.

**Table 3:** Results of the orthogonal-design L_9_(3^4^) test

Test	A	B	C/°C	D/min	Size	EE/%
1	55∶40 (1)	1:10 (1)	60 (1)	12 (1)	146.9	80.21
2	55∶40 (1)	1:15 (2)	50 (2)	24 (2)	173.0	76.32
3	55∶40 (1)	1:20 (3)	40 (3)	36 (3)	184.4	61.25
4	65∶30 (2)	1:10 (1)	50 (2)	36 (3)	152.2	81.26
5	65∶30 (2)	1:15 (2)	40 (3)	12 (1)	172.6	73.27
6	65∶30 (2)	1:20 (3)	60 (1)	24 (2)	197.4	68.92
7	75∶20 (3)	1:10 (1)	40 (3)	24 (2)	159.1	76.37
8	75∶20 (3)	1:15 (2)	60 (1)	36 (3)	186.0	70.12
9	75∶20 (3)	1:20 (3)	50 (2)	12 (1)	213.1	64.93
K1	168.10	152.73	176.77	177.53		
K2	174.07	177.20	179.43	176.50		
K3	186.08	198.30	172.03	174.20		
R	17.97	45.57	7.40	3.33

**Table 4:** Analysis of variance

Factor	*S*_*T*_	df	*F*	Significance
A	502.40	2	28.76	*
B	3130.15	2	178.61	*
C	84.28	2	4.82	
D	17.47	2	1.00	
E (error)	17.47	2		

*p<0.05

**Fig. 3:**
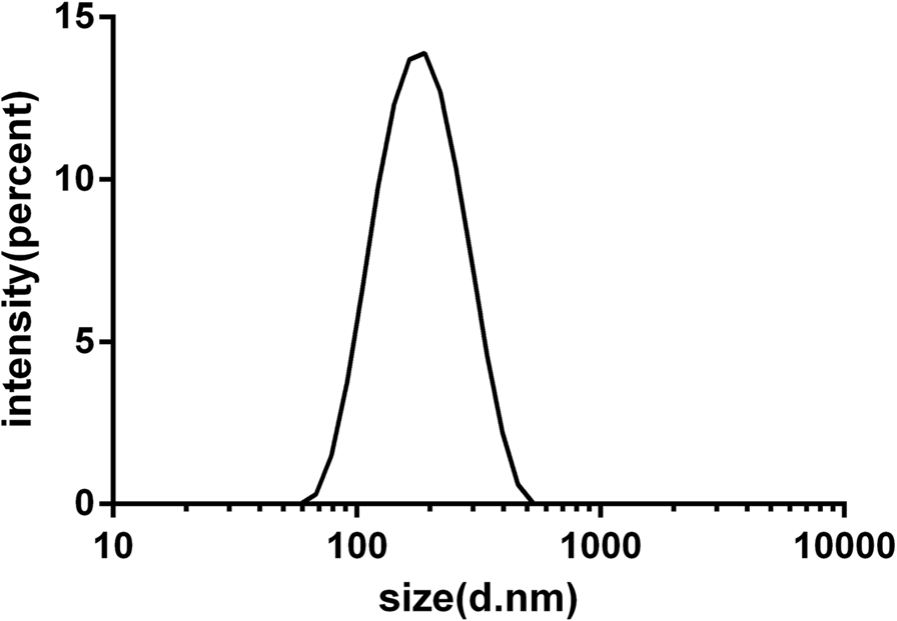
Particle size distribution of DLCL

**Fig. 4:**
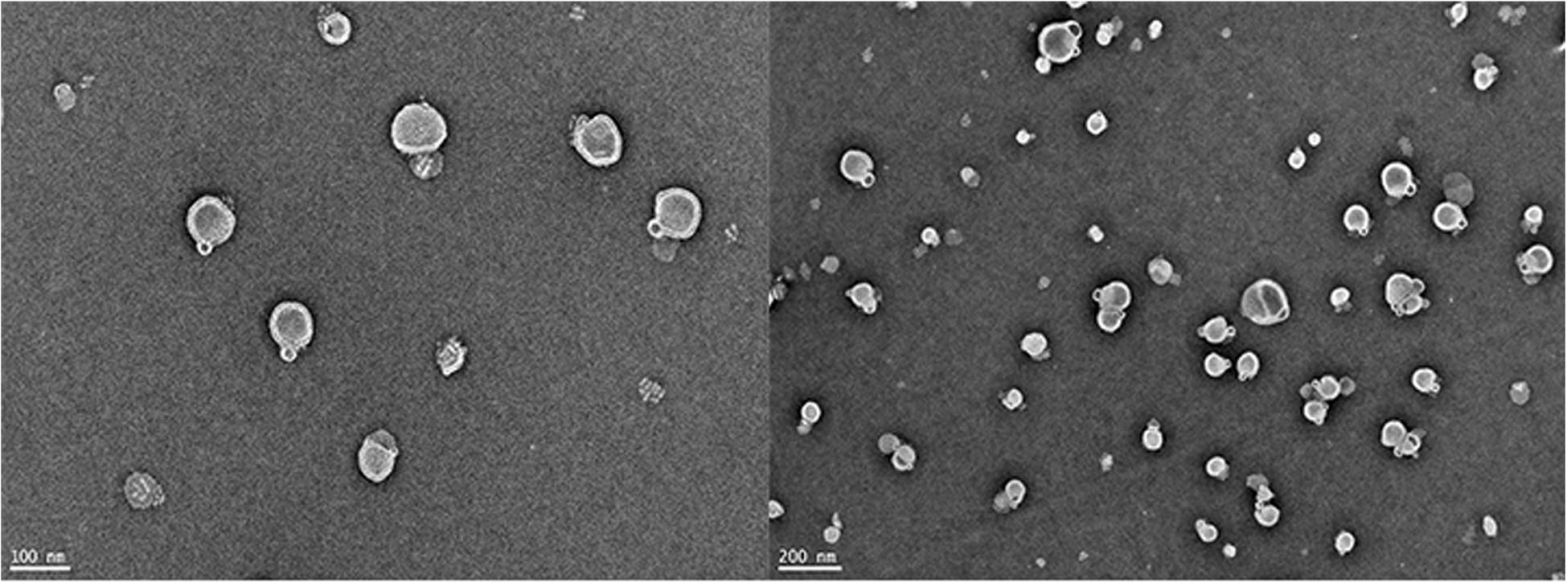
TEM photograph of DLCL

Three batches of DLCL were prepared in parallel according to the above-optimized process. The EE was 85.3 ± 3.6%, and the drug loading was 8.2 ± 1.4%. The average particle size was 156.1 ± 3.0 nm with the PDI of 0.294 ± 0.012 and the zeta potential of − 49 ± 0.6 mV. The particle size distribution of DLCL was shown in Fig. 3, which indicated that under the optimized preparation process conditions, the prepared DLCL had a narrow and uniform particle size distribution. The shape and structure of the DLCL particles observed by TEM was round or elliptical, and the size was basically uniform (Fig. 4).

Daidzein is a typical drug with poor hydrophilic and lipophilic properties. The directional combination of the drug to the polar end of the phospholipid can make them both in a highly dispersed state. The crystal characteristics of the drug are inhibited, and the lipid solubility was increased [11]. It was reported that in the interaction between daidzein and lipid bilayer, about 15% of daidzein is located in the hydrophilic region of the liposome membrane [23, 24] and the rest is distributed at the water/membrane interface [25]. According to the results of TEM, the double structure of DLCL was obvious, and the insertion of daidzein did not affect the double structure of lipids.

### Effect of Preparation Processes on In Vitro Drug Release

**Fig. 5:**
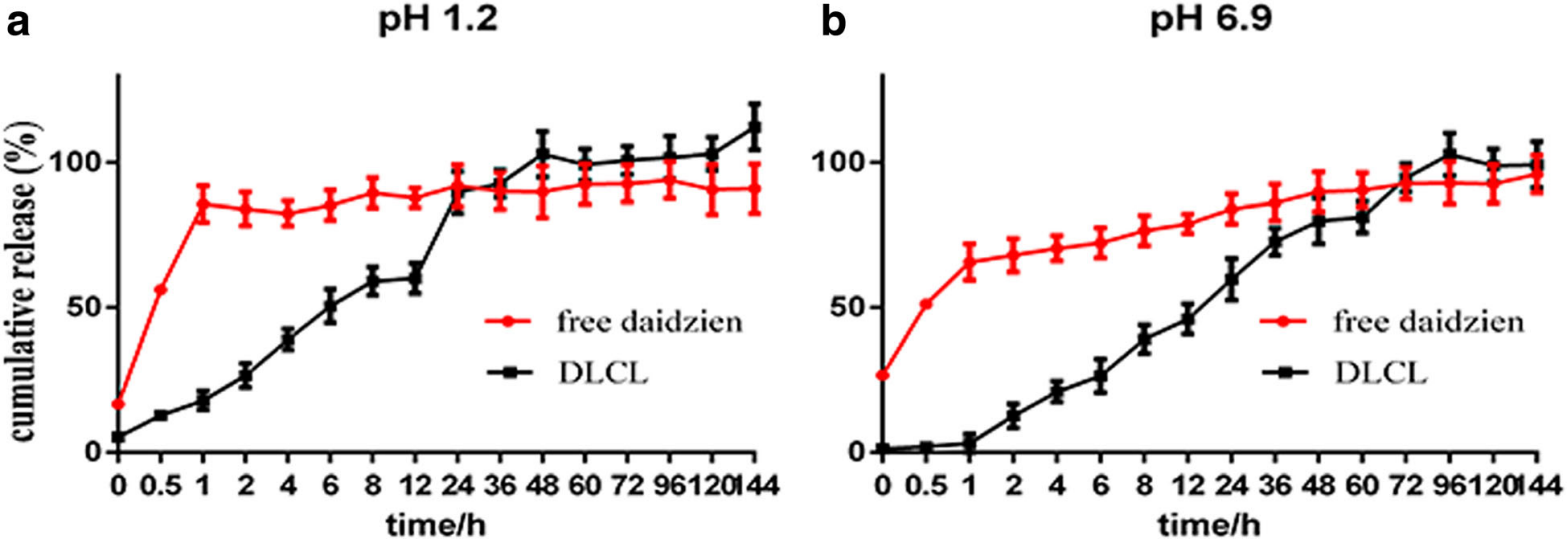
In vitro release of DLCL and free daidzein at hydrochloride solution (pH 1.2) containing 0.5% Tween 80 (**a**) and phosphate buffer (pH 6.9) containing 0.5% Tween 80 (**b**) (mean ± SD, *n* = 3)

The results of in vitro release experiments were shown in Fig. 5. In the release medium of the simulated gastric fluid (0.1 mol/L HCL containing 0.5% Tween-80), the cumulative release rate of daidzein was about 85% at 1 h and complete release at 12 h; DLCL released 18% at 1 h, 60% at 12 h, and 100% at 48 h. In the release medium of simulated intestinal fluid (25 mM PBS buffer containing 0.5% Tween-80, pH 6.9), the cumulative release rate of daidzein was about 73% at 1 h, 84% at 12 h, and complete release at 24 h; DLCL released 3% at 1 h, 59% at 12 h, and 100% at 48 h.

The results of in vitro release experiment showed that DLCL was significantly slow-release in dialysis media with pH 1.2 and pH 6.9, and the release rate in dialysis media with pH 1.2 was faster than that in dialysis media with pH 6.9. This may be due to the fact that lipid bilayer structures are vulnerable under acidic conditions and have poor stability.

### Effect of Preparation Processes on the In Vivo Pharmacokinetics

**Fig. 6:**
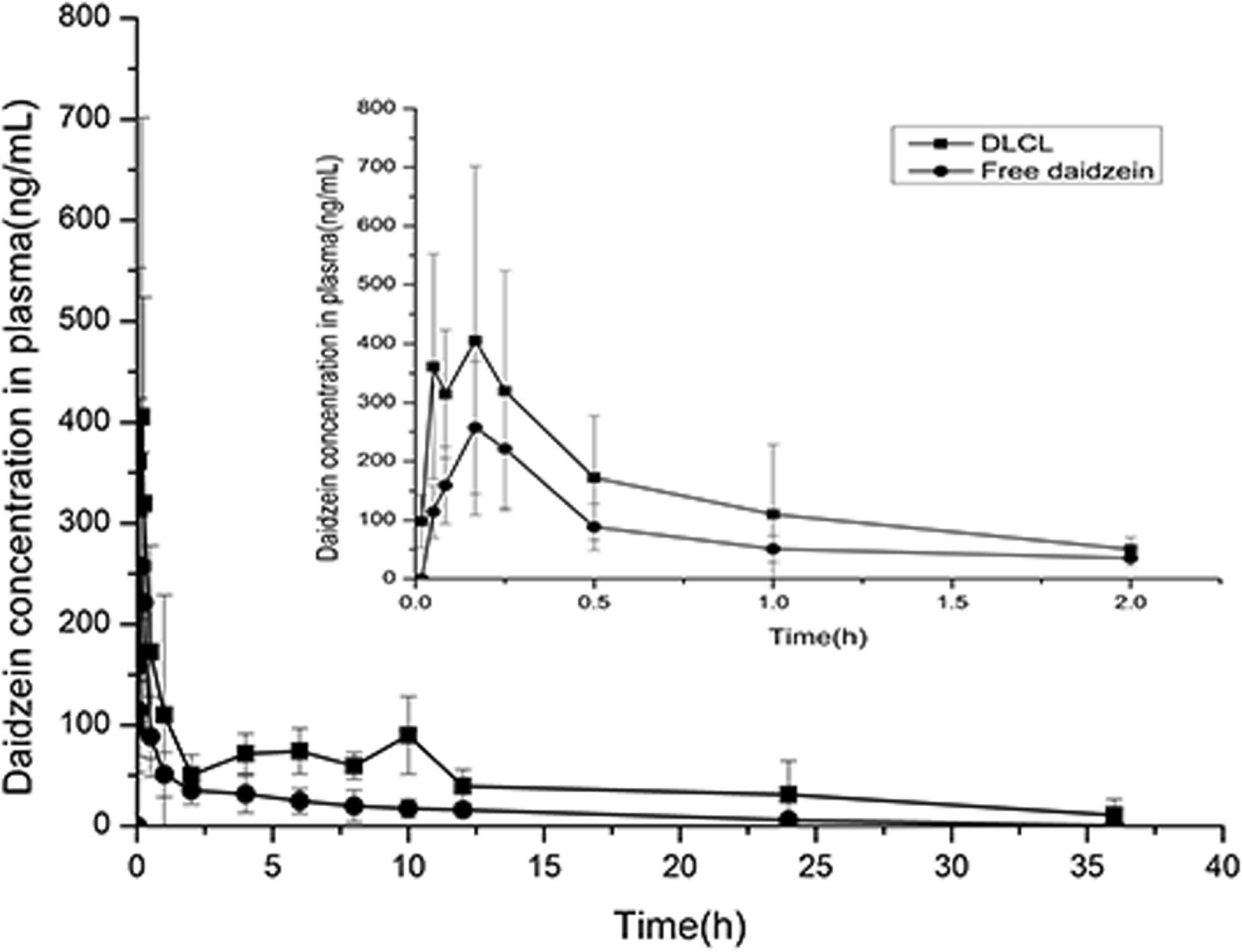
Mean plasma concentration-time curves of DLCL and free daidzein after oral administration of a single dose of daidzein (30 mg/kg) (mean ± SD, *n* = 5)

The mean plasma concentration-time curves of daidzein after oral administration of a single dose of daidzein and DLCL were shown in Fig. 6, and the main non-compartmental pharmacokinetic parameters of daidzein in both groups were displayed in Table 5. The results showed that under the iso-dose daidazein (30 mg/kg) in both groups, the plasma concentration of daidzein in DLCL group was always higher than that in daidzein group. The AUC_0−*t*_ (the area under the plasma drug concentration-time curve, which represents the total absorption after a single dose and reflects the drug absorption degree) of daidzein in DLCL group was 1515.52 ± 532.40 μg/L*h, which was 2.5 times than that of the daidzein group (*p* <  0.05). Additionally, the MRT_0−*t*_ (mean residence time, which is the time required for the elimination of 63.2% of drug in the body) and *t*_1/2_ (the elimination half-life, which is the time required to halve the plasma drug concentration of the terminal phase) of daidzein in DLCL group were prolonged by 1.6 times and 1.8 times than that of daidzein group, respectively (*p* <  0.05). The pharmacokinetic results indicated that DLCL could not only reduce the first-pass effect of daidzein to promote its oral absorption, but also prolong its mean resident time to achieve the slow-release effect.

**Table 5:** Main pharmacokinetic parameters of daidzein after rats were orally administered with DLCL and daidzein (mean ± SD, *n* = 5)

Parameter	Unit	Daidzein group	DLCL group
AUC_0−*t*_	μg/L*h	647.95 ± 166.43*	1515.52 ± 532.40
AUC_0−*∞*_	μg/L*h	650.33 ± 166.53*	1606.64 ± 554.69
MRT_0−*t*_	h	7.81 ± 1.12*	12.51 ± 2.63
*t*_1/2_	h	4.83 ± 1.06*	8.70 ± 3.342
*T*_max_	h	0.20 ± 0.045*	0.12 ± 0.053
*C*_max_	μg/L*h	316.39 ± 87.76	355.05 ± 38.96

*p < 0.05 vs daidzein group

## Conclusions

It has been reported that nano-lipid carriers, including self-emulsifying drug delivery system (SEDDS), solid lipid nanoparticles (SLN), and nano-structured lipid carriers (NLC), can effectively improve the solubility, permeability, gastrointestinal stability, and oral bioavailability of drugs. SEDDS is composed of anhydrous isotropic oil, emulsifier, auxiliary emulsifier, solubilizer, and drug. Through the emulsification of lipids in the gastrointestinal tract, it can increase the surface area absorption and the permeability of drugs and promote drugs to enter systemic circulation. However, SEDDS has a low drug load and is prone to drug crystallization and in vivo precipitation, and the correlation between in vitro and in vivo results is poor [26, 27]. SLN is composed of drug, lipid, and surfactant. With unique particle structure characteristics and controllable release advantages, SLN can significantly improve the targeting and promote the uptake of cancer cells when used to encapsulate anticancer drugs. However, SLN is not suitable for encapsulating hydrophilic drugs and drugs with cationic charge [28]. NLC is the second generation of lipid nanoparticles formed by a mixture of liquid and solid lipids, with higher stability than SLN. It can effectively encapsulate hydrophobic molecules and prolong the residence time of drugs in the body, but its preparation cost is high [29].

In order to improve the poor oral bioavailability of daidzein, recent studies were focused on its novel drug delivery system, such as daidzein-phospholipid complex loaded lipid nanocarriers with 91.7 ± 1.5% of EE and 6.87-fold of increased AUC [11], daidzein-self-assembled nanodelivery system with 85.9 ± 2.7% of EE ninefold of increased AUC [12], and daidzein-PLGA nanoparticles with 81.9% of EE and 5.57-fold of increased AUC [13].

In the present work, the EE and drug loading of DLCL prepared under optimized conditions were 85.3 ± 3.6% and 8.2 ± 1.4%, respectively. The in vitro complete release times of DLCL in the medium of pH 1.2 and pH 6.9 increased by four- and twofold of that of free drugs, respectively. After rats were orally administered with a single dose of daidzein (30 mg/kg) and DLCL (containing equal dose of daidzein), the *t*_1/2_, MRT_0−*t*_, and AUC_0–*t*_ of daidzein in DLCL group increased by 1.8-, 1.6-, and 2.5-fold as compared with those in daidzein group, which indicated that DLCL promoted the oral absorption and prolonged the mean resident time of daidzein in rats.

## Data Availability

The authors declare that the materials, data, and associated protocols are promptly available to the readers without undue qualifications in material transfer agreements. All data generated and analyzed during this study are included in this article.

## Abbreviations

AUC: Area under the time-concentration curve;
*C*_max_: Peak concentration
DL tube: Desolvent tube
DLCL: Daidzein long-circulating liposomes
DSPE-mPEG2000: Distearoyl phosphoethanolamine-PEG2000
EE: Encapsulation efficiency
ESI: Electrospray ionization
HPLC: High performance liquid chromatography
IS: Internal standard
LC-MS/MS: Liquid chromatography-tandem mass spectrometry
*m*/*z*: Mass-to-charge ratio
MPS: Mononuclear phagocyte system
MRM: Multi-reaction monitoring mode
MRT: Mean residence time
MS: Mass spectrogtaphy
PBS: Phosphate buffered saline
PDI: Polydispersity index
PEG: Poly ethylene glycol
RSD: Relative standard derivation;
SD: Standard deviation
SEM: Standard error of mean
SPC: Phosphatidylcholine
*t*_1/2_: Elimination half-life
TEM: Transmission electron microscope
*T*_max_: The time of peak concentration

## References

[1] Qin Y, Shu F, Zeng Y, Meng X, Wang B, Diao L, Wang L, Wan J, Zhu J, Wang J, Mi M (2014). Daidzein supplementation decreases serum triglyceride and uric acid concentrations in hypercholesterolemic adults with the effect on triglycerides being greater in those with the GA compared with the GG genotype of ESR-β RsaI. J Nutr.

[2] Casanova M, You L, Gaido KW, Archibequeengle S, Janszen DB, Heck HA (1999). Developmental effects of dietary phytoestrogens in Sprague-Dawley rats and interactions of genistein and daidzein with rat estrogen receptors alpha and beta in vitro. Toxicol Sci.

[3] Fonseca D, Ward WE (2004). Daidzein together with high calcium preserve bone mass and biomechanical strength at multiple sites in ovariectomized mice. BONE.

[4] Guo JM, Xiao BX, Liu DH, Grant M, Zhang S, Lai YF, Guo YB, Liu Q (2004). Biphasic effect of daidzein on cell growth of human colon cancer cells. Food Chem Toxicol.

[5] Chinta SJ, Ganesan A, Reis-Rodrigues P, Lithgow GJ, Andersen JK (2013). anti-inflammatory role of the isoflavone diadzein in lipopolysaccharide-stimulated microglia: implications for Parkinson’s disease. Neurotox Res.

[6] Setchell KD, Faughnan MT, Zimmer NL, Brown NM, Wolfe BE, Brashear WT, Desai P, Oldfield MF, Botting NP, Cassidy A (2003). Comparing the pharmacokinetics of daidzein and genistein with the use of 13C-labeled tracers in premenopausal women. Am J Clin Nutr.

[7] Doerge DR, Chang HC, Churchwell MI, Holder CL (2000). Analysis of soy isoflavone conjugation in vitro and in human blood using liquid chromatography-mass spectrometry. Drug Metab Dispos.

[8] Kulling SE, Honig DM, Simat TJ, Metzler M (2000). Oxidative in vitro metabolism of the soy phytoestrogens daidzein and genistein. J Agr Food Chem.

[9] Rowland I, Faughnan M, Hoey L, Wähälä K, Williamson G, Cassidy A, Rowland I (2003). Bioavailability of phyto-oestrogens. Brit J Nutr.

[10] Setchell K, Brown NP, Zimmer-Nechemias L, Wolfe B, Brashear W, Kirschner A, Cassidy A, Heubi J (2001). Bioavailability of pure isoflavones in healthy humans and analysis of commercial soy isoflavone supplements. J Nutr.

[11] Zhang Z, Huang Y, Gao F, Bu H, Gu W, Li Y (2011). Daidzein-phospholipid complex loaded lipid nanocarriers improved oral absorption: in vitro characteristics and in vivo behavior in rats. Nanoscale.

[12] Zhiwen Z, Yan H, Fang G, Zhiwei G, Huihui B, Wangwen G, Yaping L (2011). A self-assembled nanodelivery system enhances the oral bioavailability of daidzein: in vitro characteristics and in vivo performance. Nanomedicine-Uk.

[13] Ma Y, Zhao X, Li J, Shen Q (2012). The comparison of different daidzein-PLGA nanoparticles in increasing its oral bioavailability. Int J Nanomedicine.

[14] Bozzuto G, Molinari A (2015) Liposomes as nanomedical devices. Int J Nanomedicine 975 10.2147/IJN.S68861 PMC4324542 25678787

[15] Kulkarni SB, Betageri GV, Singh M (1995). Factors affecting microencapsulation of drugs in liposomes. J Microencapsul.

[16] Pattni BS, Chupin VV, Torchilin VP (2015). New developments in liposomal drug delivery. Chem Rev.

[17] Allen TM, Chonn A (1987). Large unilamellar liposomes with low uptake into the reticuloendothelial system. FEBS Lett.

[18] Ishida T, Harashima H, Kiwada H (2001). Interactions of liposomes with cells in vitro and in vivo: opsonins and receptors. Curr Drug Metab.

[19] Jae Sun L, Youn HS, Lee EK (2015). Imaging-based analysis of liposome internalization to macrophage cells: effects of liposome size and surface modification with PEG moiety. Colloids Surf B Biointerfaces.

[20] Allen TM, Cullis PR (2013). Liposomal drug delivery systems: from concept to clinical applications. Adv Drug Deliv Rev.

[21] Wang XH, Cai LL, Zhang XY, Deng LY, Zheng H, Deng CY, Wen JL, Zhao X, Wei YQ, Chen LJ (2011). Improved solubility and pharmacokinetics of PEGylated liposomal honokiol and human plasma protein binding ability of honokiol. Int J Pharm.

[22] Saw PE, Park J, Lee E, Ahn S, Lee J, Kim H, Kim J, Choi M, Farokhzad OC, Jon S (2015). Effect of PEG pairing on the efficiency of cancer-targeting liposomes. THERANOSTICS.

[23] Lehtonen JYA, Adlercreutz H, Kinnunen PKJ (1996). Binding of daidzein to liposomes. Biochim Biophys Acta.

[24] Mohit R, Yuriy Z, Venable RM, Pastor RW, Nagle JF, Stephanie TN (2012). Structure and elasticity of lipid membranes with genistein and daidzein bioflavinoids using X-ray scattering and MD simulations. J Phys Chem B.

[25] Dwiecki K, Neunert G, Polewski P, Polewski K (2009). Antioxidant activity of daidzein, a natural antioxidant, and its spectroscopic properties in organic solvents and phosphatidylcholine liposomes. J Photochem Photobiol B.

[26] Kamla P, Smita R (2015). Oral bioavailability: issues and solutions via nanoformulations. Clin Pharmacokinet.

[27] Chatterjee B, Hamed AS, Ahmed MDA, Mandal UK, Sengupta P (2016). Controversies with self-emulsifying drug delivery system from pharmacokinetic point of view. Drug Deliv.

[28] Lin CH, Chen CH, Lin ZC, Fang JY (2017). Recent advances in oral delivery of drugs and bioactive natural products using solid lipid nanoparticles as the carriers. J Food Drug Analysis.

[29] Gaba B, Fazil M, Ali A, Baboota S, Sahni JK, Ali J (2015). Nanostructured lipid (NLCs) carriers as a bioavailability enhancement tool for oral administration. Drug Deliv.

